# A retrospective clinical study on the resin infiltration of proximal caries lesions: the operator’s effect

**DOI:** 10.1007/s40368-021-00653-y

**Published:** 2021-09-25

**Authors:** E. Diab, D. Hesse, C. C. Bonifacio

**Affiliations:** Department of Paediatric Dentistry, Academic Centre for Dentistry Amsterdam (ACTA), Gustav Mahlerlaan 3004, 1081 LA Amsterdam, The Netherlands

**Keywords:** Caries infiltrant, Dental caries, Education, Synthetic resins

## Abstract

**Purpose:**

This retrospective university-based study investigated the effect of operators’ training and previous experience on the success of resin infiltration (RI) in arresting proximal non-cavitated caries lesions in primary and permanent teeth.

**Methods:**

Information was collected regarding RI of proximal non-cavitated caries lesions in primary and permanent teeth with a follow-up period up to 32 months. Factors investigated were: operators’ clinical experience and training, patient’s age, tooth, arch, mouth-side, surface treated, tooth separation, and baseline lesion depth. Kaplan–Meier survival and Cox regression analysis with shared frailty were used (*α* = 5%).

**Results:**

A total of 130 proximal surfaces treated on 115 teeth of 43 children (11 ± 4.4 years) were evaluated. Survival of RI was 46% up to 32 months. Lesions treated by non-trained dentists were more likely-to-present progression than those performed by non-trained dental students under supervision (HR 2.41, 95% CI: 1.00–5.80); conversely, no difference was found between non-trained dental students under supervision and trained dentists (HR 0.52, 95% CI: 0.16–1.70*).* Additionally, dentin lesions were 59% more-likely-to-present progression than enamel lesions (HR 0.41, 95% CI: 0.17–0.99).

**Conclusion:**

The operator’s experience and training could influence the success of RI on proximal non-cavitated caries lesions and it should be taken into consideration when choosing this treatment modality.

## Introduction

The management of caries lesions nowadays follows the concepts of the minimal invasive philosophy as the traditional operative invasive methods have been substituted by less invasive approaches (Frencken et al. 2012). In this way, the treatment of non-cavitated caries lesions preferably comprises of strategies to enhance remineralisation, e.g. fluoride therapy or improving the individual's oral hygiene (Cury and Tenuta 2009; Holmen et al. 1987). Although those methods can halt the progression of incipient lesions, their effectiveness relies on patient compliance. Therefore, the success of those methods is often jeopardized by the poor adherence to dental home preventive and non-invasive measures by individuals (Ratledge et al. 2011), especially when focused on proximal surfaces (Ashkenazi et al. 2012).

In this manner, a micro-invasive approach was created aiming to bridge the gap between non-invasive and invasive treatments (Phark et al. 2009) The so-called resin infiltration (RI) of caries lesions is a strategy in which a low-viscosity resin is used to fill the porosities present in the initial stages of caries lesions development, creating a diffusion barrier against cariogenic challenge (Meyer-Lueckel and Paris 2008). As a result, the lesion structures are strengthened and cavitation is prevented, without sacrificing sound tooth structure (Paris et al. 2011). In fact, three recent systematic reviews concluded that the treatment of non-cavitated enamel and initial dentinal proximal caries lesions using RI was more effective than professional fluoride application or advice on flossing (Dorri et al. 2015; Domejean et al. 2015; Chatzimarkou et al. 2018).

Despite those promising results, it must be highlighted that the application of RI on proximal caries lesions is a very sensitive technique. The use of rubber dam is mandatory during its application, to protect the soft tissues from the etching gel (15% hydrochloric gel) and to guarantee a dry environment when using the hydrophobic low-viscosity resin (Paris et al. 2010). On the top of that, the treatment with RI comprises of several steps, which need to be strictly followed, to assure that the beneficial properties of the material are delivered to the patient. Therefore, most of the clinical trials regarding the use of RI were performed in university settings, by trained operators under standardised and controlled conditions (Meyer-Lueckel et al. 2012; Ekstrand et al. 2010; Martignon et al. 2012). One exception is a recent investigation that aimed to evaluate the efficacy of RI of proximal caries lesions that were treated in private practice setting by practitioners that followed a one-day training session including treatments on simulation units. The authors concluded that the use of RI is an efficacious method to prevent the progression of initial proximal lesions. Hence, RI should be considered as a viable alternative to be applied on a daily basis, as no differences were detected on operator level, even though the investigators did not check the adherence of dentists to the RI protocol (Meyer-Lueckel et al. 2016). Still, the literature lacks on studies comparing the different levels of clinical experience and training of the operators performing RI on proximal caries lesions. Therefore, in this retrospective university-based study, we investigated the effect of operators’ training and prior experience on success of RI to arrest the progression of proximal non-cavitated caries lesions in primary and permanent teeth. The hypothesis raised is that the progression of infiltrated proximal lesions would not be influenced by the different clinical experience and training levels of the operators.

## Materials and methods

The present study has been exempted from oversight by the Local Research Ethics Committee (protocol number #2018067, Academic Center for Dentistry Amsterdam—ACTA, The Netherlands). The target population was children from 4 to 17 years old that sought treatment at ACTA during the period between 2010 and 2013, when they were treated by operators with different clinical experience and training levels. All the information employed in this study was gathered from electronical clinical records and personal information of the patients was kept confidential. To be eligible to participate into this study, children should have at least one proximal non-cavitated caries lesion on a primary or permanent molar treated with low-viscosity RI (Icon, DMG, Hamburg, Germany). Radiographically, the lesions were evaluated according to the score system proposed by Mejàre et al. (1998) (Table 1) and only lesions presenting a radiolucency ranging from the outer half of enamel (score 1) up to radiolucency into dentin, but without obvious spread in the dentin (score 3) were included in this study. Additionally, the patient’s file should contain at least one radiograph made before the treatment (baseline) and another one, made at a minimum follow-up interval of 6 months post-treatment. Finally, only radiographs of good quality were considered, meaning that they should allow good visibility of the enamel and dentin, without overlap on the proximal surfaces.

**Table 1:** Score system to classify caries lesion depth radiographically (Mejàre et al. 1998)

Code	Radiograph extention of the caries lesion
0	No visible radiolocency
1	Radiolucency in outer half of enamel
2	Radiolucency in the inner half of enamel
3	Radiolucency into dentin, but without obvious spread in the dentin
4	Radiolucency with obvious spread in the outer half of the dentin
5	Radiolucency with obvious spread in the inner half of the dentin

The operators had different clinical experience and training levels and were grouped into: (1) non-trained undergraduate dental students, supervised by clinical instructors; (2) non-trained dentists, who treated the lesions just by following the manufacturer’s instructions; (3) trained dentists, who followed a theoretical (3 h) and hands-on training on simulation units (2 h). All operators performed the treatment according to the manufacturer’s instructions.

The treatments were performed without the use of local anesthesia and always with the use of rubber dam. Tooth separation was done either by placing orthodontic elastics (ORMCO®Z-pack elastics Washington, D.C. USA) between the molars 1 week before the treatment, or using the wedge provided by the manufacturer on the day of treatment. The choice of separation technique was made according to the age, cooperation of the child and level of proximal contact strength of the region receiving RI. When the plastic wedge could not be inserted forcefully enough due to one or more aspects mentioned before, elastics were chosen. The proximal surface to be treated and adjacent tooth were cleaned with pumice and the affected proximal surface was acid-etched with 15% HCl gel (Icon, DMG, Hamburg, Germany) for 120 s. The etching was then rinsed for 30 s and dried with oil-free and water-free air. After that, the lesion was desiccated by applying ethanol (Icon, DMG, Hamburg, Germany) for 30 s, followed by air blowing again with oil-free and water-free air. The low-viscosity RI (Icon, DMG, Hamburg, Germany) was then applied and after letting it sit for three minutes, the excess of material was removed using a dental floss and the RI was light-cured from all sides for at least 40 s (total). Then low-viscosity RI was applied for the second time, and after letting it sit for 60 s, the excess of material was removed using a dental floss and the low-viscosity RI was light-cured from all sides for at least 40 s (total). Finally, the rubber dam was removed and polishing strips were used to polish the surface.

Interproximal radiographs were taken before the treatment (baseline) and on a basis of a six months interval using either interproximal tabs (Hager Werken Emmenix-Flap Bite-Wing Tabs Duisburg, Germany) or a standard interproximal holder (KerrHawe, Washington D.C., USA), according to the age and depending on the cooperation of the child. The radiograph equipment used was a Heliodent DS (Sirona Dental Systems Inc., Long Island City, USA) with 60 kV, 7 mA and a 0.12 s exposure time. The digital radiographic images were acquired using a phosphor plate detector and were processed using the digital Classic DIGORA® Optime (Soredex, Tuusula, Finland).

All children received oral hygiene instructions and diet counselling, as part of the treatment protocol. Also, children received full dental treatment according to their need. Further, a recall interval of 6 months for at least 2 years after treatment was establish for patients treated with RI. It was decided that, during each follow-up appointment, children would be evaluated clinically, as well as radiographically. This decision was based on the fact that Icon (DMG, Hamburg, Germany) was a relatively new material in 2010, and uncertainties were still present regarding the management of proximal caries lesions using this technique, especially in pediatric patients.

One independent dentist (ED) retrieved all the information from electronic dental records. The factors potentially associated with failures were investigated, including patient and treatment-related characteristics, such as operators’ clinical experience and training (non-trained undergraduate dental students, non-trained dentists or trained dentists), patient’s age (4–6, 7–12, 13–17 years of age), tooth type (primary or permanent), arch (maxilla or mandibula), mouth-site (right or left), surface treated (mesial or distal), tooth separation technique (elastic or wedge) and baseline lesion depth (enamel or dentin). The same dentist (ED) gathered the digital radiographic images from the electronic dental records of the included patients and coded them using a random numbers table. Those images were then saved as a Microsoft Powerpoint presentation; therefore, the examiners were blinded regarding the chronological order of the radiographs, as well as had no information on the operator responsible for the RI or any other patient and treatment-related characteristics. All images were standardised using the same magnification to avoid bias during the evaluation.

The main outcome of this study was treatment’s failure, defined by the progression of the lesion evaluated radiographically. Two evaluators (DH and CCB), who are specialists in pediatric dentistry, were calibrated for reproducibility according to the score system previously described (Mejàre et al. 1998). For that, the radiographs of 20 patients were evaluated at 2 different moments, with 2 weeks interval between the evaluations. In case of a discrepancy, they discussed the evaluation criteria to achieve an agreement. The efficacy of RI to arrest the progression of proximal non-cavitated caries lesions in primary and permanent teeth was evaluated by classifying caries lesion arrestment as success and caries lesion progression as failure.

Descriptive analysis was performed to provide a distribution summary according to the independent variables. Data collected from patients’ records were included in a database and statistical analysis was carried out using Stata 11.2 software (StataCorp, Texas, USA). All significant differences were detected at 95% confidence level. The weighed kappa test was applied to verify the inter- and intra-examiner agreement. A Kaplan–Meier survival analysis was used to assess the association between operators’ experience and treatment failure (caries lesion progression). Data were censored at 32 months of follow-up. Curves were also adjusted to compensate for clustering of data (more than one treatment per patient). Cox regression analysis with shared frailty was performed to verify the patient and treatment-related factors associated with caries progression.

## Results

The kappa coefficient value for the radiographic inter-reproducibility evaluation was 0.87, while intra-examiner values ranged from 0.72 to 0.89.

**Fig. 1:**
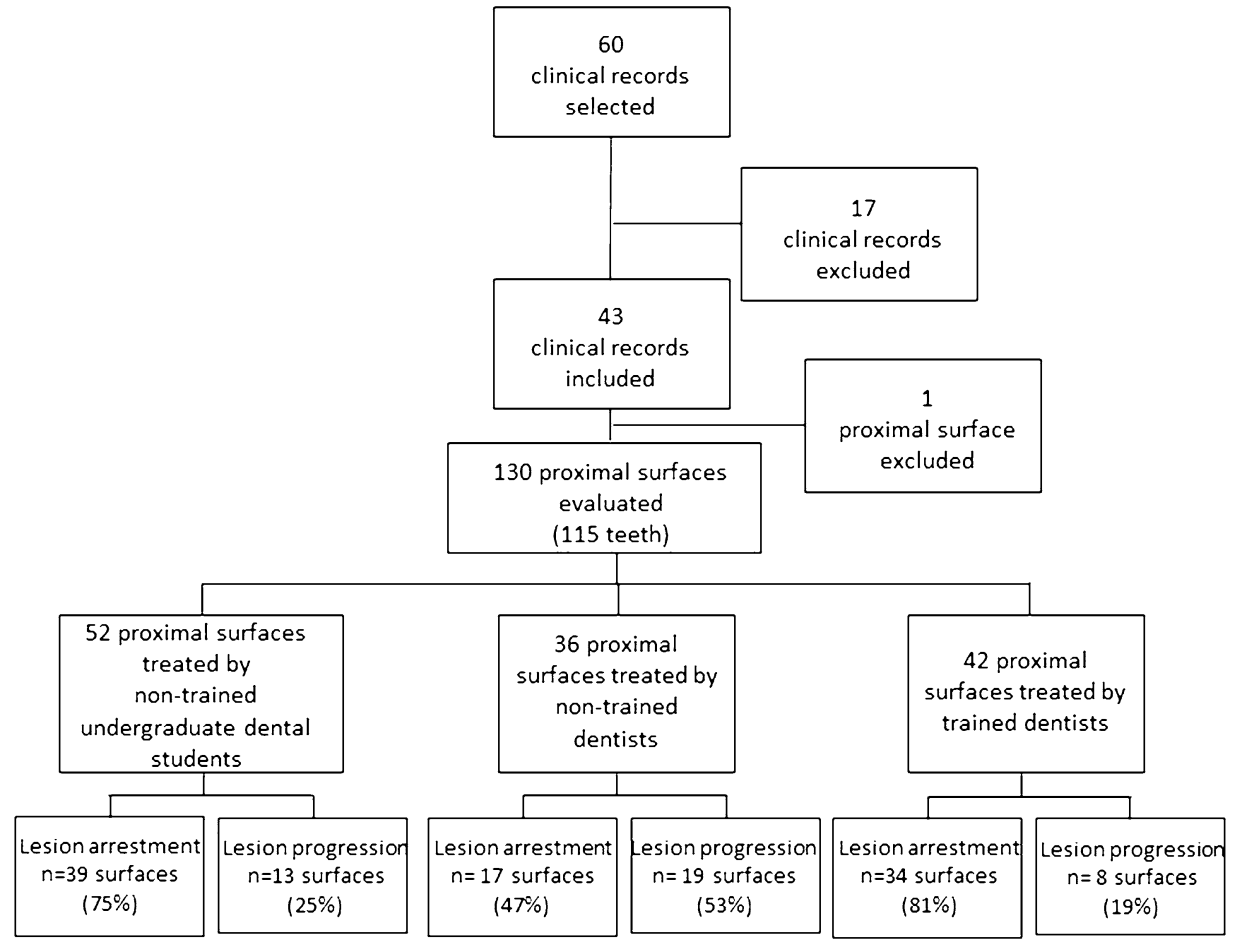
Flow diagram of the study

In this retrospective university-based study, information was retrieved from 60 dental records. From them, a total of five dental records were excluded because the baseline radiograph did not have sufficient quality for evaluation and 12 did not present a minimum 6 months of follow-up. One surface from an included child was also excluded from the analysis because at baseline the radiolucency was obviously spread in the outer half of the dentin (score 4). Finally, a total of 130 proximal surfaces on 115 teeth of 43 patients were included in the analysis (Fig. 1).

Of the 43 evaluated children, 26 (60.4%) were female and the mean age of patients was 11 years old (± 4.4). Out of the 130 surfaces evaluated, 87 (66.9%) were in the permanent teeth and 43 (33.1%) in primary molars; in 60 (46.1%) cases teeth separation was performed with elastics, while in 69 (53.1%) of cases the wedge provided by the manufacturer was used, and in one (0.8%) case the adjacent tooth was missing, so no separation was performed. We radiographically observed that 93 (71.5%) lesions were restricted to enamel when treatment was performed and 37 (28.5%) lesions were in dentin. 52 (40%) surfaces were treated by non-trained undergraduate dental students, 36 (27.7%) by the non-trained dentists, and 42 (32.3%) by trained dentists. The follow-up period ranged from 6 to 32 months. The distribution of treated surfaces, according to surface-level variables is shown in Table 2.

**Table 2:** Distribution of treatments according to surface-level variables

	Undergraduate dental students *n* (%)	Non-trained dentists *n* (%)	Trained dentists *n* (%)
Sex			
Female	34 (26.15%)	31 (23.85%)	16 (12.31%)
Male	18 (13.85%)	5 (3.85%)	26 (20.00%)
Patient’s age			
4–6 years	4 (3.07%)	13 (10.00%)	7 (5.38%)
7–12 years	25 (19.23%)	15 (11.54%)	4 (3.07%)
13–17 years	23 (17.69%)	8 (6.15%)	31 (23.85%)
Tooth type			
Primary	14 (10.77%)	22 (16.92%)	7 (5.38%)
Permanent	38 (29.23%)	14 (10.77%)	35 (26.92%)
Arch type			
Maxilla	27 (20.77%)	23 (17.69%)	17 (13.08%)
Mandibula	25 (19.23%)	13 (10.00%)	25 (19.23%)
Mouth-side			
Right	27 (20.77%)	19 (14.62%)	21 (16.15%)
Left	25 (19.23%)	17 (13.08%)	21 (16.15%)
Tooth surface			
Mesial	29 (22.31%)	23 (17.69%)	26 (20.00%)
Distal	23 (17.69%)	13 (10.00%)	16 (12.31%)
Radiographically baseline lesion depth			
Enamel	41 (31.54%)	25 (19.23%)	27 (20.77%)
Dentin	11 (8.46%)	11 (8.46%)	15 (11.54%)

*n * number of surfaces

**Fig. 2:**
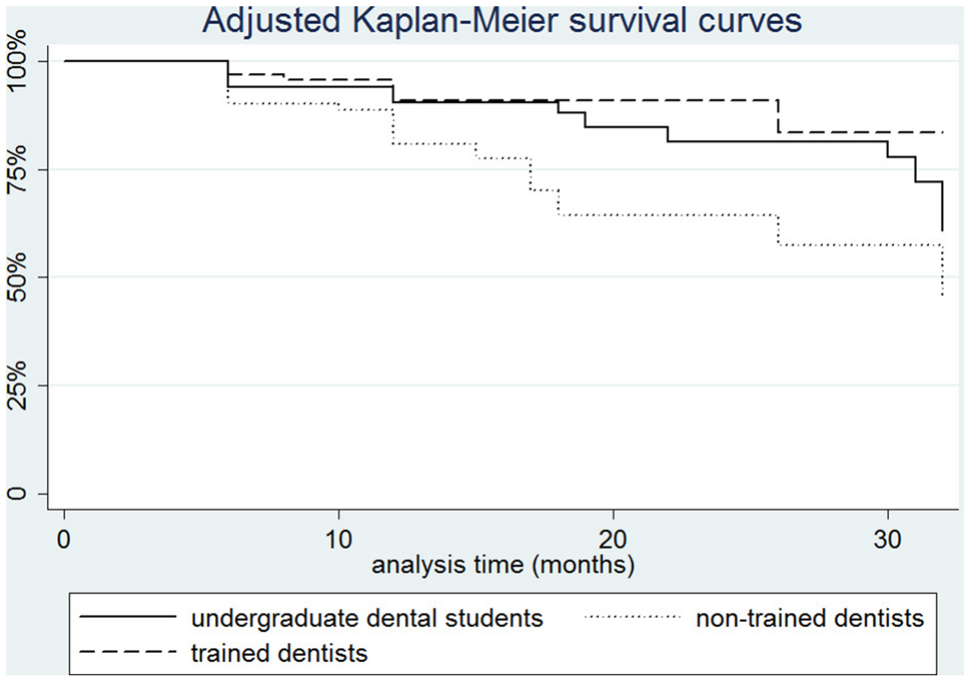
Survival curves regarding the success of RI over time by different operators’ experience and training. Log rank, *p* = *0.002*

Overall, the survival rate of treatments up to 32 months reached 46%. The survival curves, with censored data, are presented in Fig. 2. Log-rank test indicated significant difference between the different operators (Fig. 2, *p* = *0.002*).

Cox regression analysis with shared frailty showed that lesions treated by the non-trained dentists were almost 2.5-fold-more-likely-to-have caries progression than those performed by the non-trained dental students (*p* = *0.04*); however, no difference was observed between the non-trained dental students and the trained dentists (*p* = *0.28).* Additionally, the dentin lesions were 59% more-likely-to-present caries progression than the enamel lesions (Table 3).

**Table 3:** Cox regression analysis with shared frailty of treatment failures and associated factors

Variable	Crude HR 95% CI	*P*	Adjusted HR 95% CI	*P*
Operator				
Ref.: undergraduate dental students	2.36	0.05	2.41	0.04*
Non-trained dentists	0.99–5.57		1.00–5.80	
Trained dentists	0.40	0.11	0.52	0.28
	0.13–1.25		0.16–1.70	
Patient’s age				
Ref.: 4–6 years	0.92	0.87	1.55	0.62
7–12 years	0.33–2.51		0.27–8.79	
13–17 years	0.42	0.16	0.93	0.90
	0.13–1.39		0.04–92.15	
Tooth type				
Ref.: primary	0.50	0.12	0.59	0.31
Permanent	0.21–1.20		0.22–1.61	
Type of arch				
Ref.: maxilla	1.37	0.34	–	–
Mandible	0.70–2.69			
Mouth-side				
Ref.: right	0.83	0.61	–	–
Left	0.42–1.67			
Surface				
Ref.: mesial	0.93	0.84	–	–
Distal	0.48–1.80			
Separation technique				
Ref.: wedge	1.27	0.54	–	–
Elastic	0.58–2.76			
Radiographically baseline lesion depth				
Ref. Dentin	0.43	0.04	0.41	0.04*
Enamel	0.18–0.99		0.17–0.99	

*HR* Hazard ratio, *CI * confidence interval; *denotes statistical significance

## Discussion

Studies have been conducted comparing the application of RI with sealants, fluoridated varnishes, as well as with a placebo treatment on proximal non-cavitated caries lesions, and the results show that RI is more effective in arresting the progression of proximal caries lesions (Ekstrand et al. 2010; Martignon et al. 2012; Mayer-Lueckel et al. 2016; Paris et al., 2020). Despite of those promising results, the treatment with RI involves many steps, and, therefore, is considered a complex procedure (Mattos-Silveira et al. 2015). It is known that the operators’ experience and individual skills can influence the longevity of restorative treatments (Hickel et al. 2015; Demarco et al. 2012). To the best of our knowledge, however, this is the first study that compared the influence of operator’s clinical background and training on the progression of proximal non-cavitated caries lesions after treatment with RI.

The results of this retrospective study showed that treatments performed by the non-trained dentists were 2.5-fold-more-likely-to-present caries progression than the non-trained undergraduate dental students working under supervision, while no significant difference was found between the non-trained undergraduate dental students working under supervision and the trained dentists. One could speculate that treatments performed by non-trained undergraduate dental students would be more prone to failures, as quality of treatment is expected to improve with the operators’ clinical experience (Altarabulsi et al. 2013), consequently improving the treatments’ longevity (Meyer-Lueckel et al. 2016). A possible explanation for this unexpected finding is that, although the undergraduate dental students were not trained for this specific procedure, they performed all treatments under the supervision of clinical instructors. Moreover, they had long appointments with no time pressure and every single step of the treatment had to be approved before proceeding to the next step, a factor that probably assured a high-quality treatment. Consequently, we could assume that the RI technique can be performed by non-trained and non-experienced operators, as long as treatments are carried out under supervision. If supervision is not possible, prior training is necessary for dentists to successfully perform the RI technique.

An expected result found in our research is that the lesions that were located in dentin when RI was performed were 59% more-likely-to-present caries progression than the enamel lesions. This result was not surprising, as it is well known that dentin lesions are more likely to progress and tend to have a faster development when compared to enamel lesions (Mejàre et al. 1999; Pitts 1983), even when treated with RI (Martignon et al., 2012; Liang et al. 2018; Peters et al. 2019). Another relevant discussion point identified is the overall survival rate of treatments of this study. Our results showed an overall survival rate of 46% up to 32 months follow-up, which is lower when compared to the success percentages reported in previous studies (Meyer-Lueckel et al. 2012; Martignon et al. 2012; Paris et al. 2020). It is relevant to mention that these previous studies were randomised clinical trials with only trained operators, unalike the operators of the present study. Further, it is also important to indicate that in our research a survival analysis was used, and if we would take into consideration only the success of RI (absence of caries lesion progression), independently of the time in function, we would have reached a success of 70% (90/130). The Kaplan–Meier estimator takes into consideration censored data and the time of occurrence of an event, leading to an estimative that can be considered consistent with the desired simple ratio (Kishore et al. 2012; Austin 2017). Therefore, although the Kaplan–Meier estimator resulted in a lower survival rate, it can be considered a suitable statistical approach for this kind of studies.

An important discussion point is the fact that in 53.1% of the cases, teeth were separated using a plastic wedge. The wedge was used to slightly separate the teeth for the RI application. If the wedge was difficult to be inserted or if the patient reported pain during the wedge insertion, the option of using elastic separators was given and the RI treatment was postponed 1 week. This was done to avoid placing the wedges not as forcefully as needed, which would introduce bias on the RI success due to placement challenges. Furthermore, the radiograph exam was not standardised in this research and either interproximal tabs or standard interproximal holder were used. The reason for this conduct is that the age of the studied population ranged from 4 to 17 years old and sometimes the children presented uncooperative behavior, which impaired the use of radiograph holders. Still, all radiographs were taken using the same equipment, and the same exposure protocol. Additionally, the clinical records of patients were carefully retrieved, and only those containing radiographs with good quality were included in the analysis. Another relevant aspect is the fact that the two evaluators were blinded concerning any information on operator or any other patient and treatment-related characteristics, as well as regarding the chronological order of the radiographs. Consequently, the performance bias was avoided and the results reported in our investigation could be considered as a close representation of what happens in the practice-based circumstances.

Prospective double-blind clinical trials are described as the state of the art of clinical investigations, since this study design eliminates the majority of the risk factors commonly associated with the conduction of a clinical trial. However, the retrospective studies can also be a source of relevant information on the relationship between a disease and the risk factors associated to it. The absence of a control group could have been considered a limitation of this study if the aim was to compare different treatment modalities. However, as our aim was to investigate whether the operators’ experience and training influences the efficacy of RI in reducing the caries progression of proximal non-cavitated caries lesions, we believe that the results found in this investigation are of great value when choosing this treatment modality.

## Conclusion

Within the limitations of this study, we can conclude that the operators’ training and previous experience can influence the success of RI to arrest proximal non-cavitated caries lesions. This paper shows how sensitive is the RI technique and that prior training is necessary for dental professionals to successfully perform it. Also, lesions radiographically located in dentin at baseline are more likely to progress than lesions restricted to enamel, factor that should be taken into consideration when choosing this treatment modality.

## Data Availability

If needed, the corresponding author can provide details of data.

## References

[CR1] Altarabulsi MB, Alkilzy M, Splieth CH (2013). Clinical applicability of resin infiltration for proximal caries. Quintessence Int.

[CR2] Ashkenazi M, Bidoosi M, Levin L (2012). Factors associated with reduced compliance of children to dental preventive measures. Odontology.

[CR3] Austin PC (2017). A tutorial on multilevel survival analysis: methods, models and applications. Int Stat Rev.

[CR4] Chatzimarkou S, Koletsi D, Kavvadia K (2018). The effect of resin infiltration on proximal caries lesions in primary and permanent teeth. A systematic review and meta-analysis of clinical trials. J Dent.

[CR5] Cury JA, Tenuta LMA (2009). Enamel remineralization: controlling the caries disease or treating early caries lesions?. Braz Oral Res.

[CR6] Demarco FF, Corrêa MB, Cenci MS, Moraes RR, Opdam NJM (2012). Longevity of posterior composite restorations: Not only a matter of materials. Dent Mater.

[CR7] Domejean S, Ducamp R, Léger S, Holmgren C (2015). Resin infiltration of non-cavitated caries lesions: A systematic review. Med Princ Pract.

[CR8] Dorri M, Dunne SM, Walsh T, Schwendicke F (2015). Micro-invasive interventions for managing proximal dental decay in primary and permanent teeth. Cochrane Database Syst Rev.

[CR9] Ekstrand KR, Bakhshandeh A, Martignon S (2010). Treatment of proximal superficial caries lesions on primary molar teeth with resin infiltration and fluoride varnish versus fluoride varnish only: Efficacy after 1 year. Caries Res.

[CR10] Frencken JE, Peters MC, Manton DJ, Leal SC, Gordan VV, Eden E (2012). Minimal intervention dentistry for managing dental caries - A review: Report of a FDI task group. Int Dent J.

[CR11] Hickel R, Kaaden C, Paschos E, Buerkle V, García-Godoy F, Manhart J (2015). Longevity of occlusally-stressed restorations in posterior primary teeth. Am J Dent.

[CR12] Holmen L, Thylstrup A, Artun J (1987). Clinical and histological features observed during arrestment of active enamel carious lesions in vivo. Caries Res.

[CR13] Kishore J, Goel M, Khanna P (2012). Understanding survival analysis: Kaplan-Meier estimate. Int J Ayurveda Res.

[CR14] Liang Y, Deng Z, Dai X, Tian J, Zhao W (2018). Micro-invasive interventions for managing non-cavitated proximal caries of different depths: a systematic review and meta-analysis. Clin Oral Investig.

[CR15] Martignon S, Ekstrand KR, Gomez J, Lara JS, Cortes A (2012). Infiltrating/sealing proximal caries lesions: A 3-year randomized clinical trial. J Dent Res.

[CR16] Mattos-Silveira J, Floriano I, Ferreira FR, Viganó ME, Mendes FM, Braga MM (2015). Children’s discomfort may vary among different treatments for initial approximal caries lesions: Preliminary findings of a randomized controlled clinical trial. Int J Paediatr Dent.

[CR17] Mejàre I, Källestål C, Stenlund H, Johansson H (1998). Caries development from 11 to 22 years of age: a prospective radiographic study. Prevalence and distribution. Caries Res.

[CR18] Mejàre I, Källestål C, Stenlund H (1999). Incidence and progression of approximal caries from 11 to 22 years of age in sweden: a prospective radiographic study. Caries Res.

[CR19] Meyer-Lueckel H, Paris S (2008). Improved resin infiltration of natural caries lesions. J Dent Res.

[CR20] Meyer-Lueckel H, Bitter K, Paris S (2012). Randomized controlled clinical trial on proximal caries infiltration: Three-year follow-up. Caries Res.

[CR21] Meyer-Lueckel H, Balbach A, Schikowsky C, Bitter K, Paris S (2016). Pragmatic RCT on the efficacy of proximal caries infiltration. J Dent Res.

[CR22] Paris S, Dörfer CE, Meyer-Lueckel H (2010). Surface conditioning of natural enamel caries lesions in deciduous teeth in preparation for resin infiltration. J Dent.

[CR23] Paris S, Bitter K, Naumann M, Dörfer CE, Meyer-Lueckel H (2011). Resin infiltration of proximal caries lesions differing in ICDAS codes. Eur J Oral Sci.

[CR24] Paris S, Bitter K, Krois J, Meyer-Lueckel H (2020). Seven-year-efficacy of proximal caries infiltration—randomized clinical trial. J Dent.

[CR25] Peters MV, Hopkins AR, Zhu L, Yu Q (2019). Efficacy of proximal resin infiltration on caries inhibition: results from a 3-year randomized controlled clinical trial. J Dent Res.

[CR26] Phark JH, Duarte S, Meyer-Lueckel H, Paris S, Caries I (2009). Caries infiltration with resins: a novel treatment option for interproximal caries. Compend Contin Educ Dent.

[CR27] Pitts NB (1983). Monitoring of caries progression in permanent and primary posterior approximal enamel by bitewing radiography A review. Community Dent Oral Epidemiol.

[CR28] Ratledge DK, Kidd EA, Beighton D (2011). A clinical and microbiological study of approximal carious lesions: part 1: the relationship between cavitation, radiographic lesion depth, the site-specific gingival index and the level of infection of the dentine. Caries Res.

